# Synchronous peritoneal carcinomatosis from a buccal squamous cell carcinoma: a case report focusing on possible metastatic mechanisms and novel therapeutic modalities

**DOI:** 10.3332/ecancer.2019.954

**Published:** 2019-07-29

**Authors:** Frank S Fan, Chung-Fan Yang

**Affiliations:** 1 Section of Haematology and Oncology, Department of Medicine, Ministry of Health and Welfare Changhua Hospital, 80, Sec. 2, Chung-Jeng Rd, Pu-Shin Township, Chang-Hua County, 51341, Taiwan; 2 Department of Pathology, Ministry of Health and Welfare Changhua Hospital, 80, Sec. 2, Chung-Jeng Rd, Pu-Shin Township, Chang-Hua County, 51341, Taiwan; a https://orcid.org/0000-0002-8123-6941; b https://orcid.org/0000-0002-7366-4380

**Keywords:** peritoneal carcinomatosis, buccal cancer, squamous cell carcinoma, metastatic mechanism, CD44, CD36

## Abstract

A 53-year-old male patient was diagnosed with squamous cell carcinoma of buccal mucosa with synchronous diffuse peritoneal carcinomatosis, a very rare presentation for oral cancer. His disease was highly resistant to intensive systemic chemotherapy and progressed rapidly. So far as we know, there were only five cases with peritoneal involvement by metastatic head and neck cancer reported prior to this patient in the English literature. Immunohistochemistry study revealed that tumour specimens from both oral cavity and peritoneum were negative for tumour necrosis factor alpha and CD24 but positive for CD44 and CD36. These four molecules have been disclosed to be involved in the process of peritoneal metastasis from ovarian cancer. Their roles in the metastatic pathway and possible therapeutic policy targeting at them will be thoroughly discussed.

## Introduction

Cancer metastasis to the peritoneal cavity is not an uncommon phenomenon. According to a study based on the Swedish Family-Cancer Database containing 179,581 patients with metastatic cancer, the most common primary sites of peritoneal metastasis are ovary, breast and other genital organs in females; liver and prostate in males; colorectum, pancreas and stomach in both genders [1]. The incidence of peritoneal metastasis from a primary site in the upper aerodigestive tract is, however, extremely rare. An analysis of the National Cancer Registry of Ireland database also shows that over three quarters of the extra-abdominal primary sites for peritoneal metastasis are breast, lung and melanoma without mention of head and neck cancer [2].

In this case report, we present a middle-aged male patient with synchronous extensive peritoneal carcinomatosis from a squamous cell carcinoma in the oral cavity at the time of diagnosis. To the best of our knowledge, there are only five cases with peritoneal cavity metastasis from squamous cell head and neck cancer reported previously in the English literature, four from hypopharynx and one from larynx, respectively [3–6]. We did a small study to investigate the possible mechanisms of this unusual type of metastasis with immunohistochemical staining focusing on four important molecules recently revealed to be involved in the process of peritoneal metastasis from ovarian cancer. Our purpose is to see whether a squamous cell carcinoma could adopt similar mechanisms in constituting its metastatic pathway.

## Case presentation

A 53-year-old man was brought to our emergency unit with the chief complaint of abdomen fullness for 3 days in October 2018. There was no fever, vomiting, diarrhoea or constipation. He was a hepatitis B virus carrier and had a long history of hypertension without regular control. Alcohol abuse, cigarette smoking and betel nut chewing were all denied by himself. On physical examination, the abdomen was distended with detectable normal bowel sounds. Rebounding pain and muscle guarding were absent.

Laboratory data were remarkable for a thrombocytosis and a leucocytosis with a shift-to-left phenomenon: white cell count 17,400/μl, haemoglobin 14.3, g/dl, platelet count 490,000/μl, segments 72%, lymphocytes 6%, band forms 9%, metamyelocytes 3% and myelocytes 3%. His liver and renal functions were within normal limits but the hypersensitive C-reactive protein level was ultrahigh: 37.262 mg/dl (normal 0 ~ 0.748).

**Figure 1.**
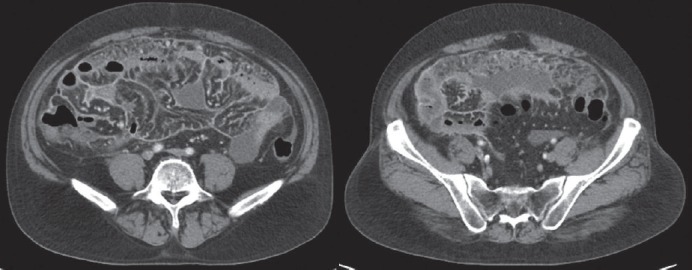
CT scan showing extensive dirty thickening of the peritoneum, fatty stranding and ascites accumulation in the abdomen cavity.

No obvious active lung lesions were noted in the chest X-ray routine film but somewhat haziness shadow was detected over the central abdomen in the kidney-ureter-bladder film. A computed tomography (CT) disclosed ascites accumulation in addition to dirty thickening and heterogeneous fat stranding of peritoneum and mesentery (Figure 1). There were no tumours in the pancreas and biliary system.

A diagnostic laparoscopy was performed under the impression of tuberculous peritonitis or peritoneal carcinomatosis. After drainage of intra-abdominal abscess-like fluid, excision biopsy of peritoneal nodules was done. Study of the ascites resulted in no evidence of tuberculous infection but a suspicion of malignancy with unidentified nature. On the other hand, serum tumour marker tests did not show any abnormality for alpha foetal protein, carcinoembryonic antigen, cancer antigen 19-9 and prostate specific antigen.

Since the initial pathologic study of the peritoneum specimen was in favour of an epithelioid carcinoma, both upper gastrointestinal scopy and colonoscopy were arranged aiming at detecting a primary malignant lesion. However, only a shallow antral ulcer, moderate reflux esophagitis, mild internal haemorrhoids and several benign polyps along the whole colon and rectum were found.

**Figure 2.**
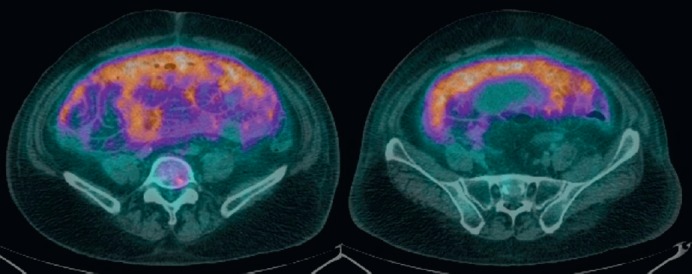
PET/CT scan showing diffuse uptake over peritoneal space (SUVmax from 5.6 to 6.4).

**Figure 3.**
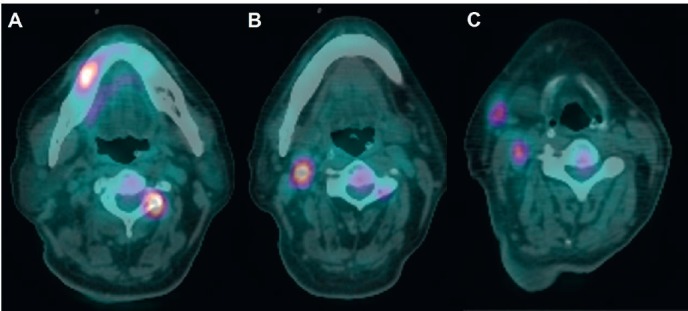
PET/CT scan. (A) A 1.8-cm lesion with moderate uptake over right lower buccal/gingival area with an avid metastatic lesion over spine. (B and C) Lymph node metastases over right neck.

**Figure 4.**
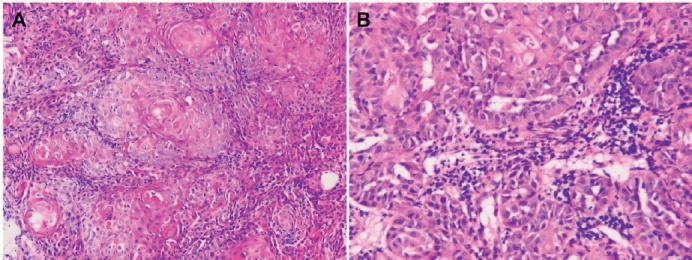
Squamous cell carcinoma of right lower buccal mucosa within oral cavity (haematoxylin and eosin stain). (A) Keratinizing large-sized polygonal tumour cells with abundant glassy/eosinophilic cytoplasm and pleomorphic nuclei, arranged in solid nests (×200). (B) Prominent acantholytic change in certain areas (×400).

The surgeon-in-charge then ordered a positron emission tomography/computed tomography (PET/CT) for discovering the hidden primary site. Much to his surprise and embarrassment, besides the predicted high uptake in the abdomen cavity (Figure 2), a 1.8-cm moderate uptake lesion over right lower buccal area appeared along with what presumed to be metastatic spots over right neck lymph nodes and vertebral bodies (Figure 3). A 2.5-cm right lower buccal ulcer with exophytic mass was subsequently disclosed by the oral surgeon and an incision biopsy led to a diagnosis of keratinizing squamous cell carcinoma (Figure 4).

**Figure 5.**
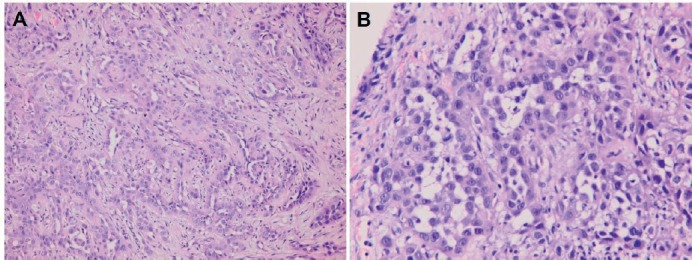
Metastatic squamous cell carcinoma over peritoneum (hematoxylin and eosin stain). (A) Neoplastic cells bearing high nucleus/cytoplasm ratio, pleomorphic nuclei and occasional nucleoli forming focal squamoid nests (×200). (B) Acantholytic change similar to that seen in buccal carcinoma specimen, resulting in glandular pattern with some detached or apoptotic tumour cells in the lumen-like structure (×400).

**Figure 6.**
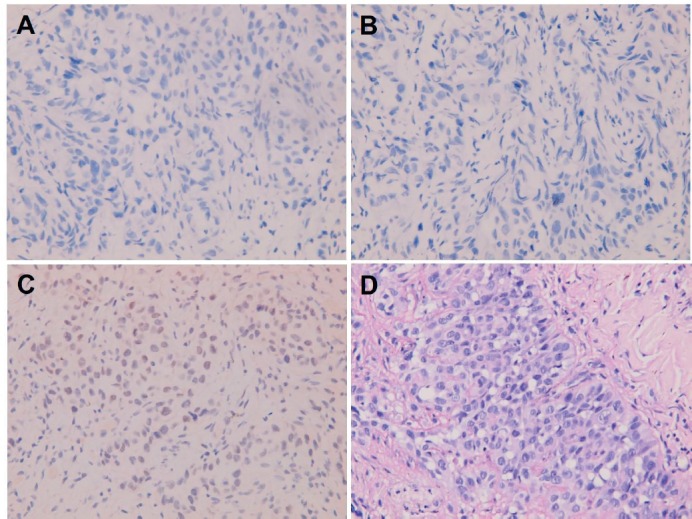
Metastatic squamous cell carcinoma over peritoneum. Negative immunohistochemical staining for (A) CK7, (B) CK20 and (C) TTF-1. (D) Negative histochemical staining for mucicarmine.

**Figure 7.**
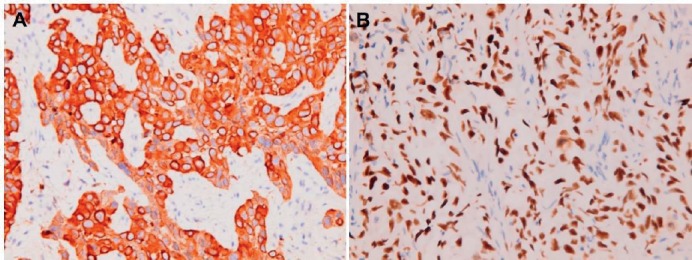
Metastatic squamous cell carcinoma over peritoneum. Positive immunohistochemical staining for (A) CK5/6 and (B) p40.

**Figure 8.**
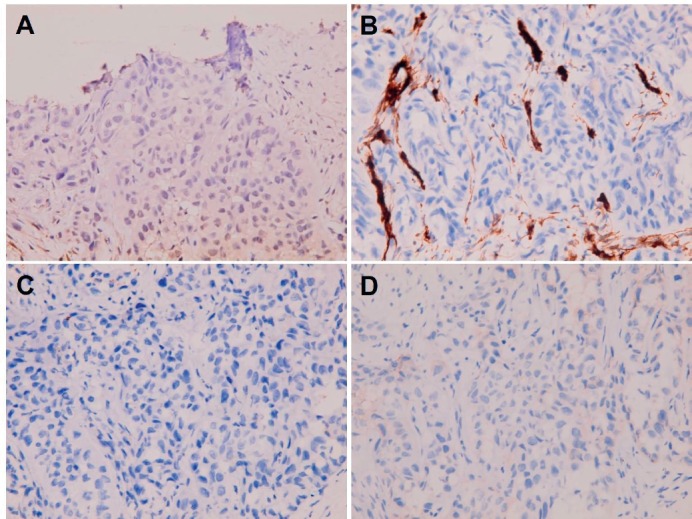
Metastatic squamous cell carcinoma over peritoneum. Negative immunohistochemical staining for (A) calretinin, (B) WT1, (C) HBME-1 and (D) D2-40.

Later on, careful investigation of the peritoneal tumour let the pathologist decide that it was most likely also a squamous cell carcinoma with focal squamoid nests and severe acantholytic change very similar to the oral cavity lesion (Figure 5). The tumour cells were negative for cytokeratin 7 (CK7), cytokeratin 20 (CK20), thyroid transcription factor-1 (TTF-1) and muscarmine (Figure 6) but positive for cytokeratin 5/6 (CK5/6) and p40 (Figure 7) in histochemistry and immunohistochemistry studies. Thus, a diagnosis of peritoneal carcinomatosis from a buccal cancer was confidently established according to modern recommendations [7, 8]. Immunohistochemistry staining for p16^INK4a^, a surrogate biomarker for human papillomavirus infection [9], was negative in both the primary and peritoneal metastatic sites. The possibility of a primary peritoneal mesothelioma could be excluded based on the negative immunohistochemical staining results for calretinin, Wilms’ tumour 1 (WT1), Hector Battifora mesothelial-1 (HBME-1) and podoplanin (D2-40) [10] (Figure 8).

After the patient’s surgical condition was over, he was transferred to the medical oncology ward for systemic chemotherapy. Intravenous cisplatin (50 mg/m^2^) and docetaxel (50 mg/m^2^) were given once, followed by continuous fluorouracil (1,000 mg/m^2^/day) for five consecutive days. This regimen was modified from what was adopted in the TAX 324 trial [11] for reducing toxicity in accordance with our past experience in treating patients who indulged in betel nut chewing, cigarette smoking, and heavy alcohol drinking. On the third day of chemotherapy, hypercalcemia was noted and the serum calcium level was immediately brought down by one dose of zoledronic acid (4 mg). Nevertheless, the tumour did not respond to chemotherapy and the patient’s general condition and conscious level deteriorated rapidly. A second course of chemotherapy with gemcitabine (1,000 mg/m^2^) and oxaliplatin (130 mg/m^2^) was given as a rescue 3 weeks after the first one. Unfortunately, there was still no response and the patient expired due to multiple organ failure.

**Figure 9.**
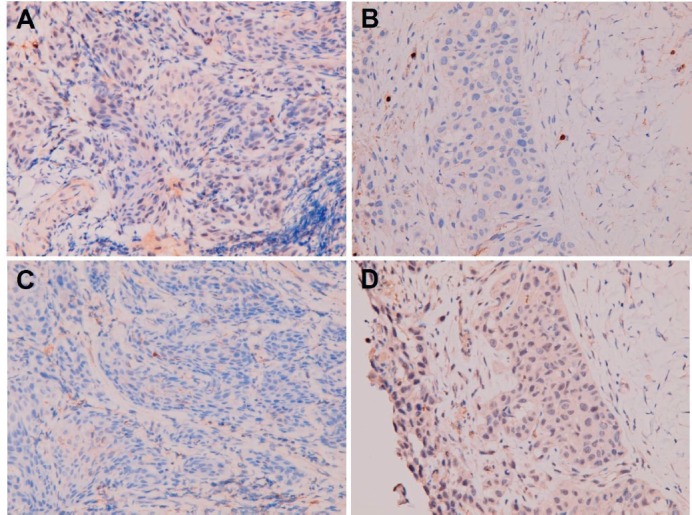
Negative immunohistochemical staining for (A, B) TNFα and (C, D) CD24 in (A and C) primary buccal squamous cell carcinoma and (B and D) metastatic squamous cell carcinoma over peritoneum.

**Figure 10.**
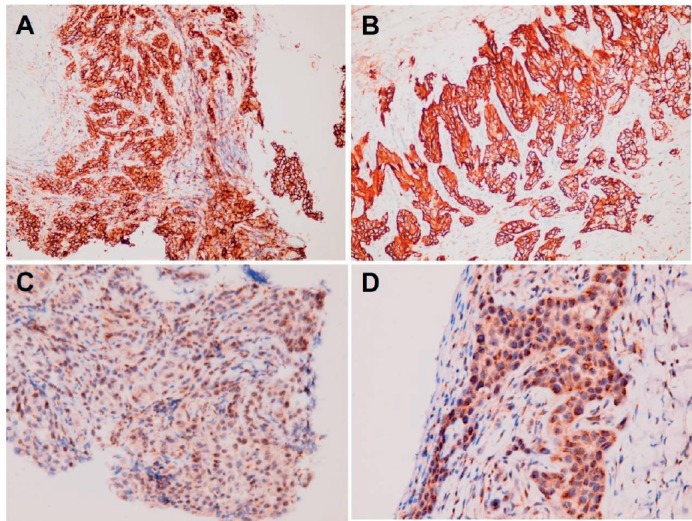
Positive immunohistochemical staining for (A, B) CD44 and(C, D) CD36 in (A and C) primary buccal squamous cell carcinoma and (B and D) metastatic squamous cell carcinoma over peritoneum.

Based on our interest in the uncommon primary site for a peritoneal carcinomatosis, immunohistochemistry study of the tumour specimens was performed to see if the tumour had adopted some characteristics antecedently found functioning in the development of peritoneal metastasis from ovarian cancer. Tumour cells from both the primary site and the peritoneum turned out to be negative for tumour necrosis factor alpha (TNFα) and CD24 (Figure 9), but positive for CD44 and CD36 (Figure 10).

## Discussion

Intraperitoneal metastasis is a very complex outgrowth containing elements of cell proliferation, epithelial-mesenchymal transition, migration, adhesion, invasion and angiogenesis. Interaction between cancer cells and target site microenvirment involves matrix metalloproteinase, inflammatory cytokines, adhesion molecules, immunosuppressors, growth factors, vascular endothelial growth factors and their receptors in making the metastatic niches relatively compatible ‘soil’ for the ‘seeds’ [12].

Ovarian cancer is notably characterised by peritoneal carcinomatosis. The cellular and molecular processes including dissemination from primary tumour, haematogenous metastasis, signalling network between cancer cells and tumour-associated immune cells in the ascites and microenvironment have been intensively investigated [13, 14]. After reviewing recent literature, we focused our interest on TNFα, CD24, CD44, and CD36 to see if these four distinct molecules previously discovered to play important roles in ovarian cancer metastasis might also be detected in this patient’s oral cancer cells.

A loop of cancer-stroma-cancer interaction in omental microenvironment has been proposed to promote peritoneal metastasis of ovarian cancer via TNFα-transforming growth factor-alpha (TGFa)-epidermal growth factor receptor (EGFR) communication [15]. As disclosed in the experiments, TNFα overexpressed and secreted by ovarian cancer cells induces TGFa expression in normal omental stromal fibroblasts through nuclear factor-κB (NF-κB) signalling. TGFa from the fibroblasts then promotes ovarian cancer colonisation in omentum through the activation of EGFR. Our patient’s oral cancer, however, does not express TNFα in either the primary site or the metastatic peritoneum, thus declining the presence of a TNFα-TNFα-EGFR loop in its metastatic process and also the possibility of using anti-TNFα agents as a therapeutic policy formerly reported to be beneficial in other cancer types [16, 17].

CD24 is a small sialoglycoprotein localised on cell surface of many tumours including ovarian cancer. It has been identified to be a cancer stem cell marker in ovarian cancer [18] and a poor prognosis predictor associated with regulation of epithelial-mesenchymal-transition which facilitates migration of cancer cells [19]. After binding to its ligand, P-selectin, CD24 exerts its function through activation of JAK2 kinase and phosphorylation of STAT3. Inhibition of JAK2 induces cytotoxicity of CD24-expressed ovarian cancer cells and increases survival of animals carrying CD24-positive tumours, supporting JAK2 as a therapeutic target for cancer cells expressing CD24 [20]. Nonetheless, our patient’s peritoneal metastatic specimen is negative for CD24, making this strategy not applicable.

CD44 is a cell membrane glycoprotein receptor with hyaluronan, a glycosaminoglycan component of the extracellular matrix, as its ligand. Upon activation, CD44 mediates and propagates self-renewal, stemness, epithelial-mesenchymal transition, migration, invasion, metastasis, and most importantly, resistance to chemotherapy, radiotherapy, endocrine and targeted therapy [21]. It has been recognised as a remarkable cancer stem cell marker for many kinds of malignancy including ovarian cancer and head and neck cancer [22]. Experiments have shown that inhibition of cancer cells’ CD44 function could reverse their malignant behaviour and sensitise them to therapy [23]. For example, antibody against CD44 was able to inhibit migration of breast cancer cells [24] and eradicate acute myeloid leukaemia stem cells [25]. Accordingly, the extraordinary chemoresistance of our patient’s carcinomatosis is thus reasonably assumed to be correlated well with its strong expression of CD44 and therapy aiming at CD44, once becoming a clinical reality, surely will provide great help for patients like ours.

CD36 is a multifunctional glycoprotein on various types of cells. It has been disclosed to be a scavenger receptor for thrombospondin-1, specific oxidised phospholipids, modified low-density lipoproteins, apoptotic cells, certain bacterial and fungal pathogens [26]. CD36 also functions in uptake of long chain fatty acid and activates signal transduction upon binding with fatty acid [27]. In this regard, exogenous fatty acid absorption has been proved capable of promoting breast cancer cell growth via CD36 [28]. On the other hand, omental adipocytes were able to fuel gastric cancer cells and probably enhanced omentum metastasis through upregulation of CD36 [29].

Furthermore, rather than only an energy supply, elevated fatty acid uptake through CD36 could also stimulate epithelial-mesenchymal transition in hepatocellular carcinoma and causatively facilitate metastasis [30]. Similar findings were detected in the experiment with ovarian cancer. CD36 expression induced by adipocytes drives ovarian cancer progression and metastasis to adipocyte-rich microenvironment in peritoneum. Intraperitoneal injection of anti-CD36 antibody effectively reduce tumour burden in mouse xenograft [31]. Most excitingly, in a human oral cancer model, anti-CD36 antibody treatment completely blocked the metastatic potential of CD36-positive cancer cells in mouse [32], reminding us of the hopeful capacity of anti-CD36 monoclonal antibody in treating malignancies like our patient’s.

## Conclusion

What we have not achieved well in the field of cancer treatment is to eradicate cancer stem cells, especially in metastatic sites and overcome drug resistance. Deeper understanding about metastatic mechanisms might reveal more key molecules in cancer stemness, migration, invasion and metastasis. Novel therapeutic modalities designed for attacking targets like TNFα, CD24, CD44 and CD36 are under aggressive development. Careful survey of cancer specimens for these biomarkers is expected to be helpful in identifying targetable antigens. The study in this patient provides an example of this ambitious kind of approach albeit awaiting more clinical trials to make efficacious treatments a reality.

## Conflict of interest

The authors have no conflicts of interest.

## Funding

There is no financial support for this report.

## Consent

The patient was single. Written informed consent was obtained from the patient’s younger brother for publication of this case report and any accompanying images. A copy of the written consent is available for review by the Editor-in-Chief of this journal.
